# A randomized controlled trial study protocol of modified Mahuang-Fuzi-Xixin decoction in the treatment of patients with mild bronchial asthma during acute exacerbation

**DOI:** 10.1097/MD.0000000000021858

**Published:** 2020-08-28

**Authors:** Wenfan Gan, Qun Huang, Maojuan Wang, Jundong Wang, Yuxin Hui, Keni Zhao, Niao Huang, Tingting Liao

**Affiliations:** a Department of Respiratory Medicine, Hospital of Chengdu University of Traditional Chinese Medicine; b Department of Ophthalmology, Hospital of Chengdu University of Traditional Chinese Medicine; c Hospital of Chengdu University of Traditional Chinese Medicine; d Department of Gastroenterology, Hospital of Chengdu University of Traditional Chinese Medicine, Chengdu, China; e Western Sydney University, Sydney, Australia; f Department of Endocrinology, Hospital of Chengdu University of Traditional Chinese Medicine, Chengdu, China.

**Keywords:** acute exacerbation, mild bronchial asthma, modified Mahuang-Fuzi-Xixin decoction, randomized controlled trial, traditional Chinese medicine

## Abstract

**Introduction:**

These years, due to dissatisfaction with western medicine treatments, traditional Chinese medicine (TCM) becomes a main treatment for bronchial asthma patients. Lung and kidney yang deficiency syndrome is a common type of asthma and the Chinese herbal medicine formula modified Mahuang-Fuzi-Xixin (MFX) decoction is prescribed for mild bronchial asthma patients with acute exacerbation syndrome. However, there is not obvious evidence to support the efficacy and safety of modified MFX decoction the efficacy and safety to treat mild bronchial asthma and the mechanism of this disease is still unclear.

**Methods:**

A double-blind, placebo-controlled, randomized clinical trial was proposed by us. After a 10-day run-in period, 180 eligible objects will be recruited in this study. These subjects will be allocated to the experimental group or control group in a 1:1 ratio. Patients in the experimental group will take modified MFX decoction. At the same time, patients in the control group will receive a matched placebo. The budesonide inhalation powder will be used as a western medicine treatment for both groups. All subjects will receive 14 days of treatment and another 6 months of follow-up. The primary outcome is the mean change in peak expiratory flow rate from the baseline to 14 days in this research. The secondary outcome includes forced expiratory volume in one second, asthma control test score, Asthma Quality of Life Questionnaire score, curative effect of TCM syndrome, and salbutamol dosage. This trial will also explore the association between the change of immunoglobulin E and modified MFX decoction treatment. Any side effects of the treatment will be recorded.

**Discussion:**

The results of this trial will provide the evidence for the effect of modified MFX decoction in patients with mild bronchial asthma during acute exacerbation. It also will explore the mechanism of this formula in the treatment of bronchial asthma, which will provide another treatment option for patients with mild bronchial asthma.

## 1 Introduction

Bronchial asthma is a chronic airway inflammatory disease with the participation of a variety of cells, including inflammatory cells, structural cells of the airway. Clinical symptoms of this disease such as recurrent wheezing, shortness of breath, chest tightness or cough often occur at night and/or early in the morning. At present, it is estimated that there are about 300 million asthma patients in the world,^[1,2]^ including nearly 30 million patients in China. At the same time, the number of these patients is increasing year by year. The World Health Organization (WHO) estimates that the number of asthma patients will increase to 400 million by 2025 worldwide.^[3]^ From researches, the prevalence of asthma is 1% to 18% worldwide.^[4]^ The researchers from China used a multi-stage random cluster sampling method to investigate the prevalence of asthma among permanent residents over 14 years old in 8 provinces (cities) in China from February 2010 to August 2011. The results demonstrated that the prevalence rate of asthma in people over 14 years old in China was 1.24%.^[5]^ At present, the prevalence of asthma is increasing and the economic burden caused by the disease accounts for about 1% of the global total burden.^[6]^ The great pressure for physical and mental health of patients might have an impact on their daily life. Therefore, asthma has become a serious global health problem. At present, inhaled corticosteroids, long-acting β-receptor agonists, theophylline sustained-release tablets, leukotriene regulators and other drugs are mainly used in the treatment of asthma in western medicine.

However, it is difficult to control the occurrence of bronchial asthma. Therefore, new treatment plan is urgently needed to improve the overall control and prognosis of bronchial asthma.

Traditional Chinese medicine (TCM) not only has the characteristics of multi-target and omni-direction in the treatment of bronchial asthma, it also plays an irreplaceable role in the adjuvant treatment of bronchial asthma. According the theory of TCM, the occurrence of asthma is related to the lung, spleen and kidney. The lung, spleen and kidney have the function of regulating the pathway of water (dredging and regulating the pathway of water metabolism). It can induce asthma if the dispersing and descending function of the lung is impaired, water overflowing due to kidney deficiency (a pathological change characterized by deficiency of kidney yang with impaired water metabolism and resultant retention of fluid).^[7]^ The essence of asthma is the dysfunction of lung, spleen and kidney.^[8]^ A great number of researches have confirmed that TCM prescription is effective as an adjuvant treatment for bronchial asthma. Wang et al. confirmed that the application of Bu-Yi-Li-Fei decoction combined with aerosol inhalation in treating elderly patients with acute attack of Bronchial asthma can reduce the airway inflammatory level, improve lung function, and relieve clinical symptoms in a short period of time.^[9]^ Cai et al. used the modified Ma-Xing-Shi-Gan decoction combined with montelukast sodium in treating bronchial asthma for children.^[10]^ Then, this research concluded that this prescription could significantly improve the lung function of children. At the same time, this formula can improve the effect of treatment and have good safety. Li and Yuedi found that the additional application of aerosol inhalation of Xiao’er Pingchuan prescription based on salbutamol sulphate aerosol could reduce the severity of asthma, control its attack and improve lung function.^[11]^ Wen-zhao did a clinical trial, the results of that showed that for the treatment of cough variant asthma, modified Xiaochaihu decoction combined with western medicine is safer and more effective, which is worthy of clinical application.^[12]^ The research of Shenhua confirmed that Wenyang Sanyu Pingchuan Recipe could reduce the levels of p65 and STAT6 in lung tissue, thus affecting the secretion of inflammatory factors IL-4, IL-13 and IFN-γin lung. Wenyang Sanyu Pingchuan recipe may relieve airway inflammation in asthma model mice by regulating NF-κB and Janus kinase/signal transduction and transcription activator (JAK/STAT) signaling pathways.^[13]^ LIU et al. found that Yu-Ping-Feng-San decoction can alleviate pulmonary inflammation, inhibit mucus secretion and the expression of MUC5AC protein, and decrease the levels of cytokines such as IL-1 β, IL-6 and TNF-α through in vivo and in vitro experiments. At the same time, it can decrease the levels of proteins and genes such as NLRP3, ASC and Caspase-1 in asthmatic mice.^[14]^ Numerous studies have shown that Pingchuan Yiqi granule can obviously improve lung function and relieve acute asthma symptoms, probably by reducing inflammatory markers (such as IL-5, IL-8, IL-1 β and PGD2) to reduce the dose of SABA.^[15]^

MFX decoction is a common prescription for asthma in China. MFX decoction is a classic formula which first recorded in the *Treatise on Febrile Diseases* by Zhang Zhongjing, a famous physician in the Eastern Han Dynasty, and it has been used for nearly 2000 years. There are 3 herbs in this composition of this prescription which includes Ephedrae Herba (*Ma Huang*), Radix Aconitu Laterlis Preparata (*Fu Zi*), Asarum Heterotropoides (*Xi Xin*). Chinese researchers have done a large number of researches to confirm the efficacy of MFX decoction in the treatment of bronchial asthma. Wang et al concluded that Ephedra Herba had a significant anti-asthmatic effect through pharmacological experiments.^[16]^ Duan et al showed that different doses of Radix Aconitu Laterlis Preparata had different degrees of anti-inflammatory and analgesic effects.^[17]^ Numerous studies have shown that β-asarone in Asarum Heterotropoides volatile oil can significantly prolong the latent time of asthma attack and fall latent time after asthma attack in guinea pigs and reduce the severity of symptoms.^[18]^ Wei et al found that MFX decoction might promote the apoptosis of Th2 cells, and then inhibit the secretion of Th2 cytokines, so as to restore the balance of Th1/Th2 to inhibit the pathogenesis of asthma, which is similar to the mechanism of hormone in clinical treatment of asthma.^[19]^ Tang et al found that the serum of MFX decoction could inhibit the release of histamine from mast cells.^[20]^ These experiments provide a pharmacological basis for MFX decoction in the treatment of bronchial asthma. The use of MFX decoction in TCM has accumulated a great deal of clinical experience in the treatment of asthma. Li-hui,^[21]^ Li-li^[22]^ and Yao^[23]^ all concluded that modified MFX decoction combined with western medicine is effective in the treatment of the Hanxiao syndrome (a type of asthma due to cold-phlegm obstructing the airway, marked by dyspnea with wheezing, cough with thin mucous expectoration, fullness and oppression in the chest, whitish and slippery tongue coating, and floating tight pulse) in elderly patients with bronchial asthma, which can effectively improve all kinds of symptoms and enhance the lung function of elderly patients. The recently published Meta-analysis based on 10 randomized controlled trials show that modified MFX decoction is effective in improving ACT score, reducing EOS level and improving lung function in adult asthma.^[24]^ These clinical studies confirm that modified MFX decoction can treat bronchial asthma, but the overall quality of the literature is not enough. At present, there still lack high-quality evidence-based medicine to confirm the clinical efficacy and safety of modified MFX decoction in the treatment of bronchial asthma. Therefore, we designed this strict randomized controlled trial to evaluate the clinical efficacy and safety of the modified MFX decoction containing 8 herbs, in the treatment of bronchial asthma.

Some studies have confirmed that immunoglobulin E (IgE) plays a certain role in the occurrence and development of asthma. The main features of airway tissue and cells in allergic asthma are infiltrating eosinophils and secretion of IgE. Th2 cells re-chemotaxis and activate eosinophils and stimulate B lymphocytes to secrete IgE.^[25,26]^ Some studies have shown that modified MFX decoction can reduce the content of serum IgE.^[27]^ Therefore, it is hypothesized that modified MFX decoction may achieve the purpose of treating bronchial asthma by reducing the content of serum IgE. Therefore, this study not only use a randomized, double-blind, placebo-controlled trial to evaluate the efficacy and safety of modified MFX decoction in the treatment of bronchial asthma, but also explore the possible mechanism of this formula in the treatment of asthma from the point of view of IgE.

## 2 Methods

### 2.1 Study design

**Figure 1:**
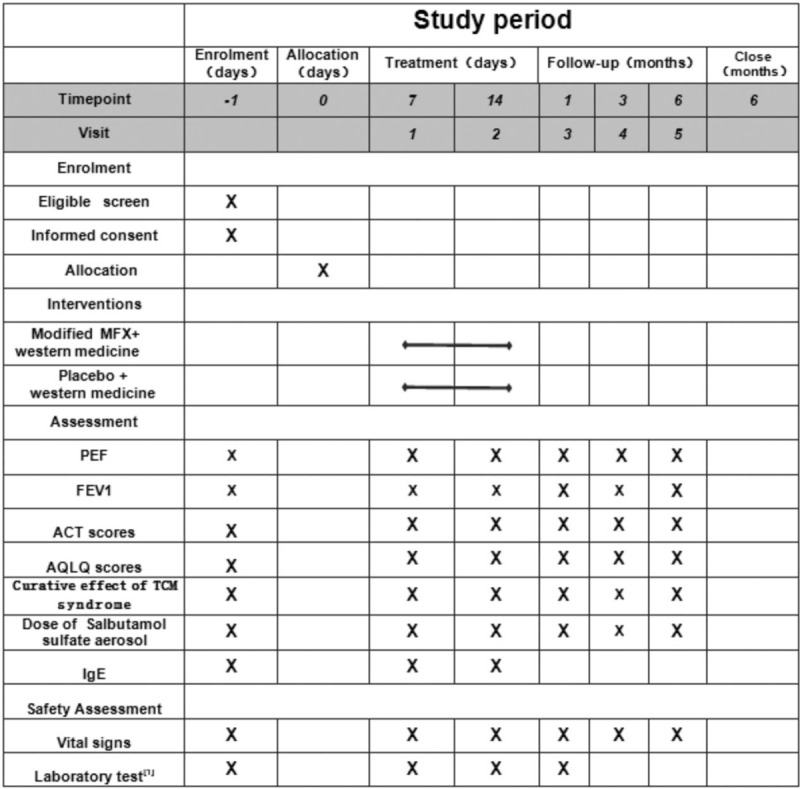
SPIRIT figure of enrollment, interventions, and assessments.^[1]^ Laboratory tests: blood, urine, feces, electrocardiogram, and kidney and liver function tests. ACT = asthma control test, AQLQ = Asthma Quality of Life Questionnaire, FEV1 = forced expiratory volume in one second, IgE = immunoglobulin E, MFX = Mahuang-Fuzi-Xixin, PEF = peak expiratory flow, TCM = traditional Chinese medicine.

**Figure 2:**
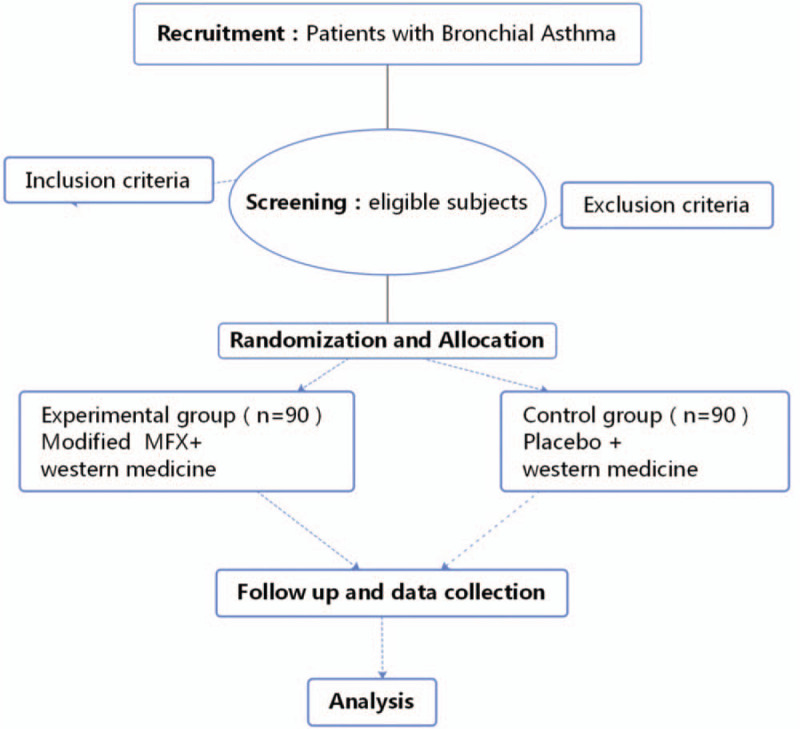
Flow chart of the study design.

This is a randomized, prospective, double-blind (patient and evaluator) placebo-controlled clinical trial. This trial has been registered with the Chinese Clinical Trial Registry (no ChiCTR2000034421, registered on July 4, 2020). This study will adhere to the Standard Protocol Items: Recommendations for Interventional Trials (SPIRIT) 2013 statement (see Fig. 1 for the SPIRIT figure of enrollment, interventions, and assessments). Figure 2 shows a diagram with the different phases of the study.

### 2.2 Ethics approval and consent to participate

The study protocol has been approved by the China Ethics Committee of Registering Clinical Trials in July 2020 and has been registered at http://www.chictr.org.cn/index.aspx with number ChiCTR2000034421 on July 4, 2020. The study will follow the ethical principles for medical research involving human subjects of the Declaration of Helsinki, adopted by the 18th General Assembly of the World Medical Association (World Medical Association, 1964), which were last revised at the association's 64th General Assembly, in Fortaleza, Brazil, in October 2013. All subjects will provide informed consent to participate.

### 2.3 Study population and recruitment

The subjects for this study will be recruited in respiratory clinics at the hospital of Chengdu University of Traditional Chinese Medicine (Chengdu, China), a national clinical trial and research center of TCM. Participants will be recruited via a local advertisement and doctor referrals. All patients referred to this service must be seen by a specialist in Department of Respiratory Medicine before receiving any treatment. And all of them will be required to write an informed consent before any procedure of this study.

### 2.4 Sample size calculation

The required calculation of the sample size was performed for primary outcome variables, peak expiratory flow rate (PEF). The statistics needed for the calculation were determined based on the study of Efficacy of the MFX decoction plus budesonide on elderly bronchial asthma of the Hanxiao syndrome and lung function. According to this study, the PEF of the control group was 303 mL/s and the standard deviation was 72 mL/s. After taking MFX decoction, the PEF in the experiment group was 276 mL/s, and the standard deviation was 68 mL/s. The following formula was employed to calculate the sample size: n = (*μ*_α_ + *μ*_β_)^2^ × (1 + 1/*k*) × *σ*^2^/(*μ*_2_ – *μ*_1_)^2^, where *σ*^2^ is overall variance, which was estimated as sample variance *s*^2^: *s*^2^ = (*s*_1_^2^ + *ks*_2_^2^)/(1 + *k*).

The proportion of participants between the experiment and control groups was set to 1:1 (*k* = 1). The study was designed to have a power of approximately 90% and two-side level of significance of 0.05 (*a* = 0.05, *β* = 0.1). *μ*_2_, *μ*_1_, *s*_1_ and *s*_2_ are mean and standard deviations in the control and experiment groups. The result is 79 per group. Thus, after assuming a follow-up loss rate of 10%, the sample size for the bronchial asthma in each group was determined to be 88, and then the total sample size would be 176. In the actual study, 90 subjects will be included in each group, with a total of 180 subjects.

### 2.5 Randomization

A list of consecutive numbers (from 1 to 180) will be generated by a member of the Sichuan TCM Evidence-Based Medicine Center before recruitment, and each number will be assigned randomly to one of the two study groups in a 1:1 ratio (Control Group, Experimental Group) using SAS 9.2 software (SAS, Cary, NC). These numbers will be placed in consecutive numbered, sealed envelopes made from carbonless paper. The envelopes will be kept by a study researcher who will not take part in the recruitment or follow-up of subjects. Without any prior knowledge of the list, a researcher, who is responsible for inclusion, will open one envelope and provide the patient with their group number on the day of inclusion. Therefore, the concealment and blinding of the assignment will be assured.

### 2.6 Binding

A double-blind clinical trial was designed in which neither the evaluators nor the subjects will be knowledgeable of their group in the study. Both modified MFX granules and placebo granules will be produced by Sichuan Green Pharmaceutical Technology Development Co, Ltd to ensure that they are identical in specifications such as appearance, shape, smell. In addition, the research team will not be allowed to talk with the patients about their group allocation. Only in emergency circumstances, like serious adverse events, can the researchers ask for advice form the principal researcher to decide whether to whether to expose blindness or not.

### 2.7 Diagnostic criteria

Subjects must meet the western medicine diagnostic criteria for bronchial asthma (Table 1) with mild acute exacerbation (Table 2)^[28]^ and the TCM syndrome diagnostic criteria of lung and kidney yang deficiency syndrome (Table 3).^[29]^ The syndrome differentiation will be determined by 2 designated deputy physicians of TCM independently.

**Table 1:**
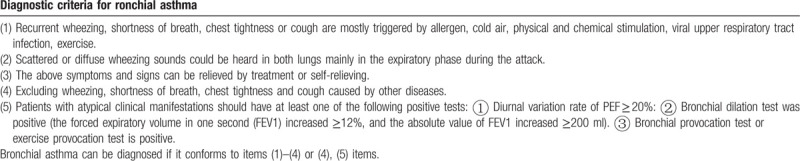
Western medicine diagnostic criteria for bronchial asthma^[28^^]^.

**Table 2:**
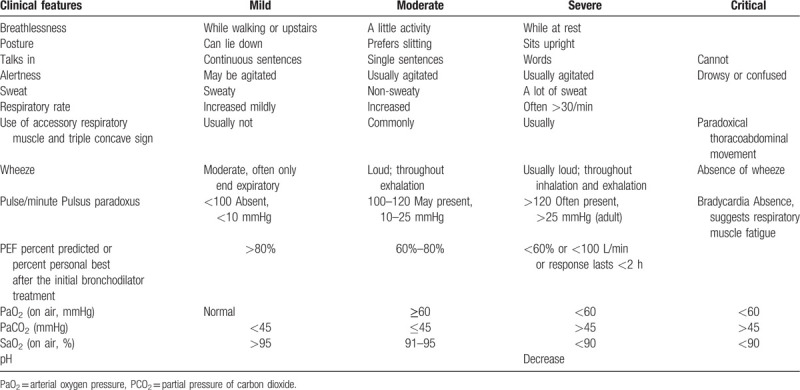
Acute asthma severity: clinical signs and symptoms.

**Table 3:**
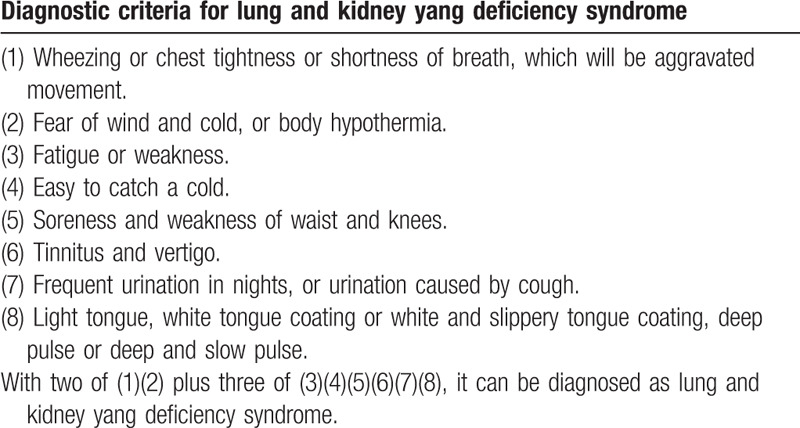
The TCM syndrome diagnostic criteria for lung and kidney yang deficiency syndrome^[29]^.

### 2.8 Inclusion criteria and exclusion criteria

The inclusion criteria will be: subjects aged 18 to 65 years, male or female; in accordance with the western medicine diagnostic criteria for bronchial asthma with mild acute exacerbation; meet the criteria for lung and kidney yang deficiency syndrome of TCM; have good ability to understand and write research-related materials, and comply with all requirements of this study voluntarily; volunteer to the study and sign the informed consent form.

The inclusion criteria will be: patients with infection of respiratory tract in 4 weeks before entering study; patients with chronic pulmonary diseases (e.g., chronic obstructive pulmonary disease, interstitial lung disease, tuberculosis and bronchiectasis); patients with severe condition of mental disorder, or laboratory test suggesting severe systemic disease (such as the dysfunction of cardio-cerebrovascular, liver and kidney, endocrine, hematopoietic system or tumor); take antibiotics within 3 months; unwilling or unable to change the current treatment plan; those who are known to be allergic to Chinese herb in the experimental drug; women who are lactating, pregnant or preparing for pregnancy; participated in other clinical studies in the past half a year.

### 2.9 Suspension and exit criteria

All subjects will get the right to exit from the clinical trial and they will get standardized treatment still if they withdraw. The reason for the exit will be recorded in their case report file (CRF). The criteria for treatment suspension and exit patients from the research will be: there are serious adverse reactions or serious complications in the course of the clinical study; the subjects have poor compliance, or use other drugs prohibited in this study; the researchers believe that continuing the study may cause serious damage to the patient's body, mind, economy; the subjects are unwilling to continue in the course of the trial, put forward a request to the researcher to suspend the clinical trial.

### 2.10 Chinese herbal medicine

The formula in experimental group is modified MFX decoction, while modified MFX mimetic agent (placebo) is the drug of control group. Both of them will be provided by the Sichuan Green Pharmaceutical Technology Development Co, Ltd (Sichuan, China). Chinese herbal medicines of each modified MFX decoction are Ephedrae Herba (Ma Huang) 10 g, Radix Aconitu Laterlis Preparata (Fu Zi) 15 g, Asarum Heterotropoides (Xi Xin) 5 g, Zingiberis Rhizoma (Gan Jiang) 5 g, Schisandrae Chinensis Fructus (Wu wei zi) 15 g, Cinnamomi Ramulus (Gui Zhi) 10 g, Scutellaria baicalensis 10 g and Glycyrrhizae Radix Et Rhizoma (Zhi Gan Cao) 3 g. The medicines in this formula are produced to granules by the manufacturer. The granules of each modified MFX decoction are divided into three parts on an average and then packaged into 3 small sachets. Placebos will be made of starch without active ingredients. With processing, the placebo will be similar with the real granules in specifications like weight, appearance, smell and taste.

## 3 Intervention

### 3.1 Treatment plan

Intervention will be provided by a physician in respiratory medicine (with more than 10 years of experience in the field of asthma therapy). According to GINA (updated 2020),^[30]^ the two groups will be treated with western medicine: Budesonide powder for inhalation (trade name: Pulmicort Turbuhaler, Specification: 0.1mg∗200 inhalation. Manufacturer: AstraZenecaAB (Sweden)), 1 inhalation each time, twice a day. Meanwhile, all patients will be acquired to use inhalers correctly with good compliance and prevent colds.

Experimental group: Subjects in the experimental group will receive modified MFX granules with a small sachet three daily for 14 days, after breakfast, lunch and dinner. The granules of each sachet will be dissolving in 100 mL warm boiled water.

Control group: Patients in the control group will be given placebo granules with a small sachet three daily for 14 days. The proceeding will be similar with the experimental group.

Concomitant asthma medications: During the trial, salbutamol sulfate aerosol (100 μg/puff, GlaxoSmithKline Pharmaceutical Co., Ltd., Suzhou) can be used as needed in case of wheezing, shortness of breath, no more than 8 puffs per day. With the requirement of the study, subjects will record the medication and the dose of it every day in their patient diary. If symptoms and signs are not relieved, the subjects will contact with the physician immediately to get further treatment according to GINA. In addition, subjects are not allowed to use other traditional Chinese medicine and other drugs for the treatment of bronchial asthma in the course of this trial.

### 3.2 Baseline data and outcomes

The baseline data of each one will be recorded at the beginning of the study. It contains the following variables: Sex, age, height, weight, work, asthma course, asthma control test (ACT) scores, and TCM syndrome scores.

Primary outcome: The primary outcome is the change in PEF (daytime and nighttime) from the baseline to the end of the treatment phase (day 15).

Secondary outcomes:

1. The forced expiratory volume in the first second (FEV1).
2. ACT scores will be used to identify the controlled situation of asthma patients.
3. Health-related quality of life: The total scores from the Asthma Quality of Life Questionnaire (AQLQ) will be used to assess the quality of life for patients in this research.
4. Curative effect of TCM syndrome.
5. Total dose of self-reported as-needed salbutamol sulfate aerosol.

Exploratory outcome: IgE will be measured by enzyme-linked immunosorbent assay.

### 3.3 Safety assessment

With recorded in *Treatise on Febrile Diseases*, one of the Four Classics of TCM, the modified MFX decoction has been used for nearly 2000 years. The dosage of Chinese herbs in this study will be complied within the People's Republic of China Pharmacopeia (2015 edition). Moreover, we will monitor the patient's vital signs and electrocardiogram (ECG), and do laboratory tests including blood, urine, stool routine, liver function, renal function to assess the safety of modified MFX decoction from the enrollment to the follow-up period.

### 3.4 Compliance of subjects

Once subjects have been grouped, researchers are responsible for follow-up to make every reasonable effort to contact the patient during the study. Compliance of subjects will be monitored and patients will be required to return all empty containers at each follow-up visit. All examination and transportation costs will be free. Doctors will explain the results of examinations to subjects every visit. Someone will remind the patient of the upcoming data collection via Wechat or the phone before visit. Additionally, treatment plan suggestion will be provided to the patients in the follow-up phase.

### 3.5 Adverse events

Any adverse events of subjects will be recorded in case report files. If a serious adverse situation occurs, the intervention will be suspended immediately. Then, we will keep an account of the detailed description about the time of occurrence, the severity of condition and the relation of test drugs. All measures will be recorded in accordance with standard operational procedures of the China Food and Drug Administration. Besides, serious adverse events will be made known to the Steering Committee and Ethics Committee within a day.

### 3.6 Statistical analysis

The data will be analyzed by the SPSS V.20.0 (Chicago, IL) software package after the collection of data from the entire sample. The significance level will be set at 0.05 (bilateral tests) and the limits of the confidence interval at 95%. A descriptive analysis of the baseline date of the sample will be performed. All the quantitative variables will be analyzed by the Kolmogorov-Smirnov test with Lilliefors corrections, to ascertain whether the variables follow a normal distribution or not. The initial homogeneity between the groups will be also analyzed (ANOVA or Kruskal-Wallis depending of the normality of data distribution).

The measurement data will be examined using group *t* tests or non-parametric tests, the count data will be tested using a chi-square test or Fisher exact probability method, and the grade data will be tested using nonparametric tests.

All data analyses will be conducted with the intention to treat principles. If there are losses of follow-up, the outcome variables that have not been recorded will be completed with the last data recorded for each subject of these data.

### 3.7 Discussion

Bronchial asthma is a serious global health problem that causes a major disease burden. Current available medications for asthma are failed to achieve satisfactory results. TCM formulas have been used in the treatment of asthma in China for a long time. Modified MFX decoction is a common prescription for this disease.

As far as we know, this is the first double-blind, placebo-controlled, randomized clinical study to confirm the efficacy and safety of modified MFX decoction plus western medicine in the treatment of bronchial asthma. The trial aims to compare the effectiveness of the western medicine alone and the western medicine plus the modified MFX decoction to treat bronchial asthma. A hundred and ninety subjects will be included in this clinical study. At the same time, they will be divided into two groups according to the randomization performed before recruitment. The variables will be evaluated post-treatment and 6 months follow-up after treatment. It will be PEF, FEV1, ACT scores, AQLQ scores, curative effect of TCM syndrome and total dose of salbutamol sulfate aerosol. In this study, we will also test the changes in IgE to explore the possible mechanism of modified MFX decoction in the treatment of asthma from the perspective of view of IgE.

If the results of the study are positive, it will be possible to give another option in the area of adjuvant treatment with bronchial asthma.

## Acknowledgments

We are grateful to the Sichuan Science and Technology Program and Science and technology development fund of Hospital of Chengdu University of traditional Chinese Medicine for funding this study.

## Author contributions

**Conceptualization:** Tingting Liao.

**Investigation:** Keni Zhao.

**Supervision:** Yuxin Hui.

**Writing – original draft:** Wenfan Gan.

**Writing – review & editing:** Qun Huang, Maojuan Wang.
